# Alprostadil attenuates LPS-induced cardiomyocyte injury by inhibiting the Wnt5a/JNK/NF-κB pathway

**DOI:** 10.1007/s00059-019-4837-0

**Published:** 2019-07-16

**Authors:** T. Yu, D. Dong, J. Guan, J. Sun, M. Guo, Q. Wang

**Affiliations:** 1 Department of Emergency, Affiliated Hospital of Qingdao University, Jiangsu Road No. 16, Qingdao, Shandong China; 2 Department of Cardiology, No. 971 Hospital of People’s Liberation Army, Minjiang Road No. 22, Qingdao, Shandong China

**Keywords:** Vasodilators, Lipopolysaccharides, Myocytes, cardiac, Cell survival, Anti-inflammatory effects, Vasodilatoren, Lipopolysaccharide, Myozyten, kardiale, Zellüberleben, Antiinflammatorische Effekte

## Abstract

**Background:**

Clinical research has demonstrated that alprostadil has an anti-inflammatory effect; however, to date, its molecular mechanisms remain unclear. This study aimed to examine the anti-inflammatory activity and related mechanisms of alprostadil in lipopolysaccharide (LPS)-treated H9c2 cells.

**Methods:**

Cell morphology was observed under an inverted light microscope, while cell viability was assessed with the 3‑(4,5-dimethylthiazolyl-2)-2,5-diphenyltetrazolium bromide (MTT) assay. Enzyme-linked immunosorbent assays (ELISA) were conducted to study biochemical indicators of cellular damage, such as released lactate dehydrase (LDH) and troponin, and inflammatory cytokine levels including interleukin-1β (IL-1β), IL-6, IL-17, and tumor necrosis factor-α (TNF-α). The mRNA expression levels of Wnt5a, c‑jun N‑terminal kinase (JNK), and nuclear factor kappa B (NF-κB) were further investigated by real-time quantitative polymerase chain reaction (RT-PCR). The effects of alprostadil on the Wnt5a/JNK/NF-κB pathway in H9c2 cells was examined by Western blotting.

**Results:**

Alprostadil increased the cell viability of LPS-stimulated H9c2 cells, reduced LDH and troponin production, and attenuated IL-1β, IL-6, IL-17, and TNF-α secretion. Moreover, alprostadil reduced the mRNA expression of Wnt5a, JNK, and NF-κB and decreased the expression of Wnt5a, NF-κB, and the ratio of p‑JNK/JNK in H9c2 cells treated with LPS. The siWnt5a or JNK inhibitor SP600125 significantly augmented the inhibitory effects of alprostadil on the Wnt5a/JNK/NF-κB pathway.

**Conclusion:**

Our results show that alprostadil has anti-inflammatory effects and could attenuate LPS-induced injury in H9c2 cardiomyocytes via the Wnt5a/JNK/NF-κB pathway.

## Abstract (de)

**Hintergrund:**

Klinische Untersuchungen haben gezeigt, dass Alprostadil einen antiinflammatoischen Effekt hat; jedoch sind die entsprechenden molekularen Mechanismen bisher nicht bekannt. Die vorliegende Studie hatte zum Ziel, die antiinflammatorische Aktivität und diesbezügliche Mechanismen von Alprostadil in Lipopolysaccharid(LPS)-behandelten H9c2-Zellen zu untersuchen.

**Methoden:**

Unter dem Lichtmikroskop wurde die Zellmorphologie untersucht, während die Lebensfähigkeit der Zellen mit dem 3‑(4,5-Dimethylthiazolyl-2)-2,5-Diphenyltetrazoliumbromid(MTT)-Assay beurteilt wurde. Enzyme-linked-Immunosorbent-Assays (ELISA) wurden durchgeführt, um biochemische Indikatoren der zellulären Schädigung zu untersuchen, z. B. freigesetzte Laktatedehydrogenase (LDH) und Troponin, sowie inflammatorische Zytokinspiegel einschließlich Interleukin-1β (IL-1β), IL-6, IL-17 und Tumornekrosefaktor-α (TNF-α). Die Werte für die mRNA-Expression von Wnt5a, c‑jun-N-terminale Kinase (JNK) und den nuklearen Faktor kappa B (NF-κB) wurden darüber hinaus durch quantitative Polymerasekettenreaktion in Echtzeit (RT-PCR) untersucht. Die Effekte von Alprostadil auf den Wnt5a/JNK/NF-κB-Signalweg in H9c2-Zellen wurden durch den Westernblottest ermittelt.

**Ergebnisse:**

Alprostadil erhöhte die Lebensfähigkeit der Zellen von LPS-stimulierten H9c2-Zellen, verminderte die Produktion von LDH und Troponin und schwächte die Sekretion von IL-1β, IL-6, IL-17 und TNF-α ab. Darüber hinaus reduzierte Alprostadil die mRNA-Expression von Wnt5a, JNK und NF-κB sowie verminderte die Expression von Wnt5a, NF-κB und das Verhältnis von p‑JNK/JNK in H9c2-Zellen, die mit LPS behandelt worden waren. Durch siWnt5a oder den JNK-Inhibitor SP600125 wurden die inhibitorischen Effekte von Alprostadil auf den Wnt5a/JNK/NF-κB-Signalweg signifikant verstärkt.

**Schlussfolgerung:**

Die vorliegenden Ergebnisse zeigen, dass Alprostadil antiinflammatorische Effekte aufweist und die LPS-induzierte Schädigung von H9c2-Kardiomyozyten über den Wnt5a/JNK/NF-κB-Signalweg abschwächen konnte.

Sepsis is a systemic inflammatory response syndrome, mainly caused by endotoxemia, a condition characterized by the presence of endotoxins in the blood. Lipopolysaccharides (LPS) are lipid-containing polysaccharides that are endotoxins and important group-specific antigens. Sepsis is often associated with multiorgan dysfunctions especially of the heart. Myocardial dysfunction, also referred to as septic cardiomyopathy (SC), is a common complication of severe sepsis. The clinical manifestation of SC is systolic and diastolic dysfunction and is often accompanied by abnormal levels of cardiac biomarkers such as troponin and natriuretic peptides (NPs) in the setting of sepsis [1]. Up to 44% of patients with sepsis showed SC [1, 2], and their mortality rate was significantly higher than those without SC [3]. Septic cardiomyopathy is a multifactorial process that involves complex interactions between the host immune system and invading pathogens. Owing to the complex clinical manifestations, numerous assessment methods, and variations in the preseptic state of the heart, there is currently no consensus about a formalized definition of SC. The specific pathogenesis of SC might be related to the injury, necrosis, and apoptosis of myocardial cells, which could be caused by inflammatory factors [4, 5]. Other studies revealed that cardiomyocyte energy metabolism disorder [6], myocardial microcirculation dysfunction, and myocardial cell hypoxia injury also played important roles in the occurrence and development of SC [7], although the molecular mechanisms and their significance in the pathogenesis of SC are still not clear.

Wnt5a predominantly activates the β‑catenin-independent Wnt signaling cascade including the Wnt/calcium and Wnt/PCP (Wnt/polarity) pathways. Recent studies showed that Wnt5a was associated with certain inflammatory disorders [8, 9]. Wnt5a could be induced by LPS in human macrophage and was found in the sera of patients with severe sepsis [10]. Some researchers thought Wnt5a was an inflammatory cytokine and could mediate the release of pro-inflammatory factors such as interleukin-6 (IL-6), tumor necrosis factor-α (TNF-α) and interferons (IFN; [11–13]). Therefore, we hypothesized that Wnt5a may play a role in LPS-induced myocardial damage. C‑jun N‑terminal kinase (JNK) is a member of the mitogen-activated protein kinase (MAPK) family and is a type of serine/threonine protein kinase. It can regulate cell growth, differentiation, proliferation, inflammation, and apoptosis by phosphorylating c‑jun or other substrates after activation [14]. The JNK signaling pathway mediates the production and release of inflammatory factors, which promotes the development of inflammation, in which the Wnt5a/JNK/nuclear factor kappa B (NF-κB) signaling cascade plays an important role [15–18]. NF-κB is an important transcription factor, as it activates several inflammatory genes after immune stimulation. The JNK signaling pathway is upstream of the NF-κB signaling pathway. Lipopolysaccharide can activate JNK pathway, promote the transfer of NF-κB to the nucleus, further increase inflammatory factors, and induce injury and apoptosis of the cells. The specific JNK inhibitor, SP600125, can significantly inhibit the activation of JNK induced by LPS and reduce the phosphorylation level of NF-κB [19–22].

Alprostadil, also known as prostaglandin E1 (PGE1), is a potent vasodilator agent. Alprostadil has numerous effects including vasodilation, protecting endothelial cells, and inhibiting the activation and aggregation of neutrophils and thrombocytes [23]. Clinical research has demonstrated that alprostadil can reduce the expression of inflammatory factors such as myeloperoxidase (MPO), TNF-α, and IL-6 in the treatment of ischemia reperfusion injury, contrast nephropathy, and diabetic nephropathy etc. [24–27]. Furthermore, PGE1 improved the myocardial microcirculation and alleviated apoptosis of myocardial cells [28, 29]. These findings indicate that alprostadil may have an anti-inflammatory effect, although its molecular mechanisms are not exactly clear. This study investigated whether alprostadil has protective effects on LPS-induced injury in myocardial cells and elucidated its potential mechanisms.

## Materials and methods

### Cell culture and treatments

The H9c2 cardiomyoblasts were obtained from the Cell Bank of Type Culture Collection of the Chinese Academy of Sciences (Shanghai, China) and cultured in Dulbecco’s modified Eagle’s medium (Invitrogen, Carlsbad, CA, USA) containing 10% fetal bovine serum (Gibco, Rockville, MD, USA) in a humidified incubator with 5% CO_2_ at 37 °C (Thermo Fisher Scientific, Waltham, MA, USA). Cells were seeded into 24-well plate and then cultured to the logarithmic growth phase (1.0 × 10^5^ cells/ml) and used for the experiment. The cells were treated with different concentrations of LPS (Sigma-Aldrich, St. Louis, MO, USA) at 10, 25, 50, 100 μg/l and/or alprostadil (Tide Pharmaceutical Co. Limited, Beijing, China) at 5 μg/l, 15 μg/l, and 45 μg/l for 3 h, 6 h, 12 h, and 24 h, respectively. The follow-up intervention concentration and time point were selected according to the results of subsequent 3‑(4,5-dimethylthiazolyl-2)-2,5-diphenyltetrazolium bromide (MTT) experiments. The siRNA interference sequence (Gima gene, Wuhan, Hubei, China) was designed according to the sequence of rat Wnt5a. Nonspecific siRNA (scramble) with non-targeting was used as a control. Cells grown to a confluence of 40–50% were transfected with siRNA using Lipofectamine 2000 reagent (Thermo Fisher Scientific, Waltham, MA, USA) according to the manufacturer’s protocol. The siWnt5a sequences were as follows: sense GGUCCCUAGGUAUGAAUAATT, antisense UUAUUCAUACCUAGGGACCTT; 5.0 µM SP600125 (Sigma-Aldrich) was used in the culture medium. The cell morphology and growth status were successively observed under an inverted light microscope (Eclipse TS100; Nikon, Tokyo, Japan).

### Cell viability assay

Cell viability assays were performed by using the MTT assay. Briefly, cells were plated into 96-well plates at a density of 5 × 10^3^ cells/well, and to each well was added 20 μl of 5 mg/ml MTT (Sigma-Aldrich) and incubated in the dark for 4 h at 37 °C. The medium was then removed, and 150 µl dimethyl sulfoxide (DMSO) was added (Sigma-Aldrich) into each well, whose absorbance values was measured at 490 nm using a Multiskan GO microplate reader (Thermo Fisher Scientific).

### ELISA detection

Commercial kits (Nanjing Jiancheng Biology Engineering Institute, China) were used to detect biochemical indicators of cellular damage such as lactate dehydrase (LDH), troponin, and the production of inflammatory cytokines, including interleukin-1β (IL-1β), IL-6, IL-17, and TNF-α, which were in the cellular supernatant and were measured with a microplate reader (Thermo Fisher Scientific) according to the manufacturer’s instruction.

### RNA extraction and RT-PCR

Total RNA of cells was extracted by using an RNA extraction kit (Invitrogen). The RNA concentration was quantified using a spectrophotometer (NanoDrop 2000; Thermo Fisher Scientific). Complementary DNA synthesis was performed by using a Prime Script RT Reagent Kit (Takara, Osaka, Japan). Quantitative real-time PCR was performed by using gene-specific primers and an SYBR Green PCR Master Mix (Takara) on a Roche Light Cycler 480 Real Time PCR System as follows: 95 °C for 30 s, then 40 cycles of 95 °C for 15 s, followed by 60 °C for 30 min. Measurements were performed in triplicates and normalized to endogenous GAPDH levels. Relative fold change in expression was calculated by using the 2‑∆∆Ct method. The sequences of primers for Wnt5a, JNK, NF-KB, and GAPDH are as follows: Wnt5a—sense TGTCTTTGGCAGGGTGAT, antisense AAGCGGTAGCCATAGTCG; JNK—sense GTTAGATGAAAGGGAGCA, antisense GCTGTCTGTATCCGAGGC; NF-KB—sense CTGCTTACGGTGGGATTG, antisense TTGCTTCGGTCTTGGTGC; GAPDH—sense ACAGCAACAGGGTGGTGGAC, antisense GAGGCAGGGATGATGTTCT.

### Western blot analysis

The cells were washed twice with pre-cooled phosphate buffered saline (PBS) at 4 °C, digested with 0.25% trypsin-EDTA solution, lysed on ice for 30 min with radio immuno-precipitation assay (RIPA) buffer and centrifuged at 12,000 *g*, 4 °C for 15 min. The supernatant was collected and the protein concentrations were quantified using the NanoDrop 2000 spectrophotometer. The same quality protein sample (25 μg) was added into the loading buffer and denatured at 100 ℃ for 5 min. When completely cooled, the proteins were separated by sodium dodecyl sulfate polyacrylamide gel electrophoresis (SDS-PAGE) and transferred to a polyvinylidene difluoride membrane (PVDF; Millipore, Billerica, MA, USA). After being blocked by 5% skim milk powder in tris-buffered saline with tween (TBST) for 2 h at room temperature, the membranes were incubated with primary antibodies against Wnt5a, JNK, p‑JNK, NF-κB, β‑actin, and Lamin B (all from Proteintech, Rosemont, IL, USA) at 4 °C for 12 h, washed three times for 5 min with TBST, then incubated with a horseradish peroxidase-conjugated secondary antibody (1:2000; Proteintech) for 2 h at room temperature, and followed by washing three times with TBST for 5 min each time. Finally, the signal was developed using an enhanced chemiluminescent kit (NCM Biotech, Suzhou, China) with the Tanon 5200 system (Shanghai, China). The optical density of each band was analyzed with the Image J 1.48 image analysis software (NIH, NY, USA), using β‑actin or Lamin B as an internal control.

### Statistical analysis

Each step of the experiment was repeated at least three times independently. The measurement data are expressed as mean ± standard deviation (SD). One-way analysis of variance (ANOVA) was used for comparison between groups. The Tukey test was used for comparison between the two groups, and SPSS 22.0 software was used for data analysis. Statistical significance was set at* p *< 0.05.

## Results

### Alprostadil increased the viability of LPS-treated H9c2 cells

**Fig. 1:**
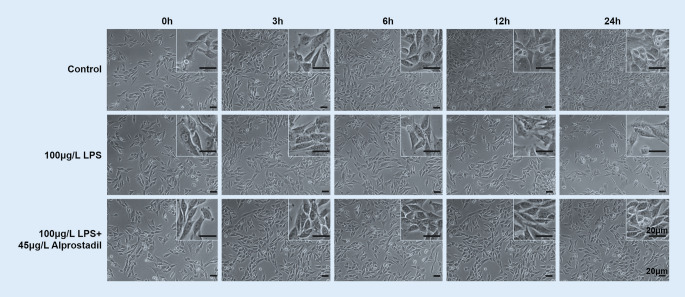
Morphological changes in H9c2 cells after treatments with 100 μg/l lipopolysaccharides (*LPS*) and 100 μg/l LPS + 45 μg/l alprostadil at different time points

**Fig. 2:**
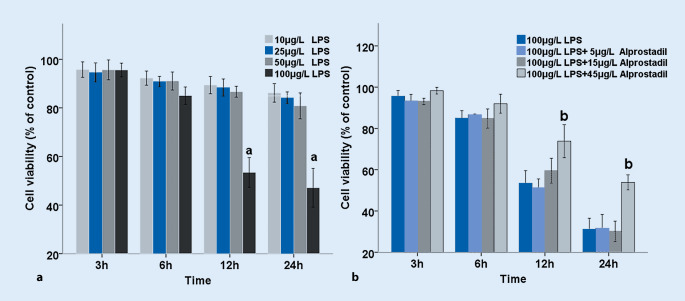
Effects of lipopolysaccharides (*LPS*) on cell viability of H9c2 cells treated by LPS with or without alprostadil. Cell viability was measured via an MTT assay, and was calculated as percentage (mean ± SD) compared with the corresponding blank control according to 490 nm absorbance. **a** H9c2 cells treated with different doses of LPS for 3 h, 6 h, 12 h, and 24 h. **b** Cells pretreated with different doses of alprostadil, then treated with 100 μg/l LPS for 3 h, 6 h, 12 h, and 24 h. Data are presented as means ± SD, and group differences were analyzed by one-way ANOVA with Tukey’s post hoc test, *n* = 3 independent experiments. *a p* < 0.01 compared with blank controls (Ctrl), *b* *p* < 0.01 compared with 100 μg/l LPS at the corresponding time point

H9c2 cells were treated with different concentrations of LPS for 0 h, 3 h, 6 h, 12 h, and 24 h. As shown in Fig. 1, it was found that LPS decreased cell proliferation with the prolongation of time. Results of the MTT assay showed that the cell viability gradually decreased with the increase in LPS concentration and time. Compared with other groups, 100 μg/l LPS had significant effects on H9c2 cell viability when treated for 12 and 24 h (*p* < 0.01; Fig. 2a); however, there was no statistically significant difference in cell viability between the 12-h and 24-h treatment. We observed effects of alprostadil on H9c2 cell activity treated with 100 μg/l LPS. Cells were pretreated with different doses of alprostadil and then treated with 100 μg/l LPS for 3 h, 6 h, 12 h, and 24 h. We found that cell viability was significantly restored by the pretreatment with alprostadil (Fig. 1). The cell viability gradually recovered in cells pretreated with 45 μg/l alprostadil for 12 and 24 h (*p* < 0.01; Fig. 2b). These results indicate that alprostadil protected H9c2 cells against LPS-induced injury; 45 μg/l alprostadil was chosen and used in the following experiments.

### Alprostadil ameliorated LPS-induced cell injury and down-regulated the expression of inflammatory cytokines

**Fig. 3:**
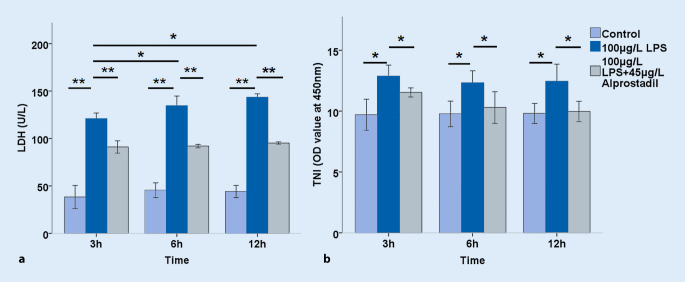
Effects of alprostadil on the release of (**a**) lactate dehydrase (*LDH*) and (**b**) troponin (*TNI*) in lipopolysaccharide (*LPS*)-treated H9c2 cells for 3 h, 6 h, and 12 h. Data are presented as means ± SD, and group differences were analyzed by one-way ANOVA with Tukey’s post hoc test, *n* = 3 independent experiments; *asterisk* *p* < 0.05, *double asterisk* *p* < 0.01

**Fig. 4:**
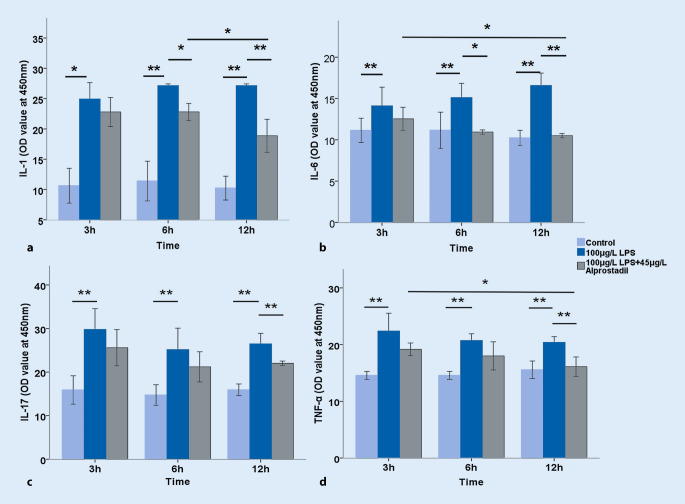
Effects of alprostadil on the levels of lipopolysaccharide (*LPS*)-induced inflammatory cytokines in H9c2 cells subjected to treatment for 3 h, 6 h, and 12 h: **a** interleukin (*IL*)-1; **b** IL-6; **c** IL-17; **d** tumor necrosis factor (*TNF*)-α. Data are presented as means ± SD, and group differences were analyzed by one-way ANOVA with Tukey’s post hoc test, *n* = 3 independent experiments; *asterisk* *p* < 0.05, *double asterisk* *p* < 0.01

Further, cell injury was examined by measuring LDH and troponin levels. It was found that LPS significantly increased the release of LDH in a time-dependent manner, which could be reversed significantly by pretreating with alprostadil (*p* < 0.01; Fig. 3a). The LPS significantly increased troponin levels in H9c2 cell supernatant, but not in a significant time-dependent manner, and the troponin level was reduced by pretreatment with alprostadil (*p* < 0.05; Fig. 3b). The effects of LPS on cytokine expression in H9c2 myocardial cells were then examined. As shown in Fig. 4, LPS significantly increased the levels of IL-1, IL-6, IL-17, and TNF-α, indicating that LPS up-regulated the expression of inflammatory cytokines, which was reversed significantly by alprostadil intervention in varying degrees. These results suggest that alprostadil ameliorated LPS-induced cell injury and down-regulated the expression of inflammatory cytokines.

### Effects of alprostadil on LPS-induced mRNA expression of Wnt5a, JNK, and NF-κB in H9c2 cardiomyocytes

**Fig. 5:**
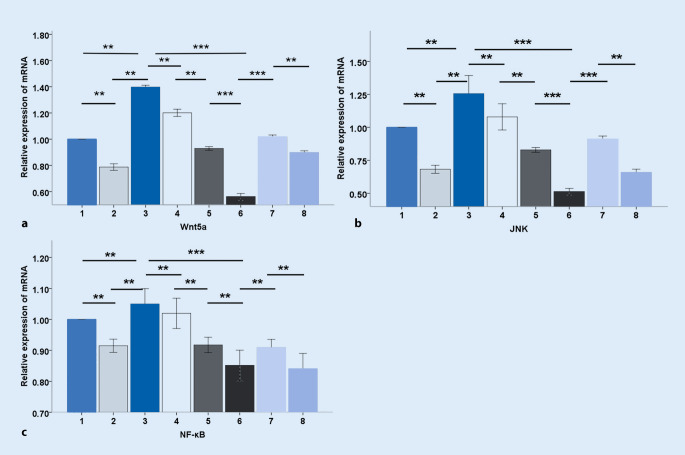
Effects of alprostadil on lipopolysaccharide (LPS)-induced mRNA expression of Wnt5a, JNK, and NF-κB in H9c2 cells. Semi-quantitative analysis of the relative mRNA expression of Wnt5a (**a**), JNK (**b**), and NF-κB (**c**). *1* control group, *2* alprostadil group, *3* LPS group, *4* LPS+alprostadil group, *5* LPS+siWnt5a group, *6* LPS+siWnt5a+alprostadil group, *7* LPS+SP600125 group, *8* LPS+SP600125+alprostadil group. Data are presented as means ± SD, and group differences were analyzed by one-way ANOVA with Tukey’s post hoc test, *n* = 3 independent experiments; *asterisk* *p* < 0.05; *double* *asterisk* *p* < 0.01; *triple* *asterisk* *p* < 0.001

To investigate the potential signaling pathways involved in LPS-induced injury in H9c2 cardiomyocytes, we first examined the relative mRNA expression in H9c2 cells treated by LPS, including Wnt5a, JNK, and NF-κB. As shown in Fig. 5, Wnt5a, JNK, and NF-κB mRNA expression was significantly elevated in response to LPS treating after 3 h (*p* < 0.01). By contrast, pretreatment with alprostadil markedly reduced their expression (*p* < 0.01). Wnt5a siRNA was then used to treat H9c2 cells. It was found that siWnt5a specifically down-regulated the expression level of Wnt5a (*p* < 0.01), while JNK and NF-κB mRNA expression was also reduced (*p* < 0.01, *p* < 0.01, respectively) after treatment with LPS. Moreover, the mRNA level of Wnt5a, JNK, and NF-κB showed a much greater significant decrease in the LPS+siWnt5a+alprostadil group (*p* < 0.001, *p* < 0.001, *p* < 0.01, respectively), indicating that alprostadil might play a role by simulating Wnt5a, and that alprostadil combined with siWnt5a can exert synergistic inhibitory effects on the mRNA expression of Wnt5a, JNK, and NF-κB.

Furthermore, H9c2 cells were treated with SP600125, an inhibitor of JNK signaling. The data showed that the mRNA expression of Wnt5a, JNK, and NF-κB were significantly decreased in the LPS+SP600125 group compared with the LPS group (*p* < 0.01, *p* < 0.01, *p* < 0.01, respectively) and their expression was further decreased in the group pretreated with alprostadil and SP600125 (*p* < 0.01, *p* < 0.01, *p* < 0.01 respectively). The mRNA expression levels of Wnt5a, JNK, and NF-κB were still high in the SP600125 group and SP600125+alprostadil group compared with the alprostadil group, siWnt5a group, and LPS+siWnt5a+alprostadil group (data not shown). Therefore, we speculated that SP600125 had an inhibitory effect on gene transcription levels of Wnt5a, JNK, and NF-κB, and that alprostadil could further enhance this inhibitory effect, but SP600125 was weaker than alprostadil and much weaker than siWnt5a.

### Alprostadil suppresses Wnt5a/JNK/NF-κB signaling in H9c2 cardiomyocytes treated with LPS

**Fig. 6:**
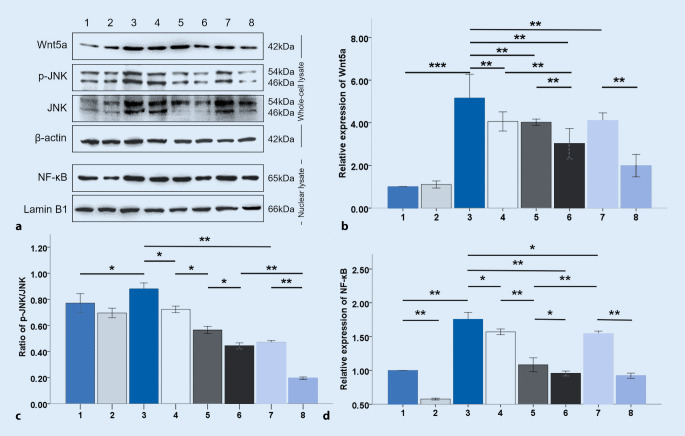
Alprostadil suppresses Wnt5a/JNK/NF-κB signaling in LPS-induced H9c2 cells. Western blot analysis was conducted using specific antibodies against Wnt5a, JNK, and NF-κB (**a**–**d**). *1* control group, *2* alprostadil group, *3* LPS group, *4* LPS+alprostadil group, *5* LPS+siWnt5a group, *6* LPS+siWnt5a+alprostadil group, *7* LPS+SP600125 group, *8* LPS+SP600125+alprostadil group. Data are presented as means ± SD, and group differences were analyzed by one-way ANOVA with Tukey’s post hoc test, *n* = 3 independent experiments; *asterisk* *p* < 0.05; *double* *asterisk* *p* < 0.01; *triple* *asterisk* *p* < 0.001

We explored the protein expression of Wnt5a, JNK, p‑JNK, and NF-κB in H9c2 cells treated with LPS by Western blotting. As shown in Fig. 6, LPS treatment remarkably increased the expression of Wnt5a, NF-κB, and the ratio of p‑JNK/JNK (*p* < 0.001, *p* < 0.01, *p* < 0.05, respectively), while pretreatment with alprostadil reduced the expression of Wnt5a, NF-κB, and the ratio of p‑JNK/JNK (*p* < 0.001, *p* < 0.05, *p* < 0.05, respectively). Next, siWnt5a was used to pretreat cells, and it was found that the expression of NF-κB (*p* < 0.01) and the ratio of p‑JNK/JNK (*p* < 0.05) were lower in the LPS+siWnt5a group than that in the LPS+alprostadil group. However, there was no significant difference in the expression of Wnt5a in the LPS+siWnt5a group and LPS+alprostadil group. Moreover, it was found that the expression of Wnt5a, NF-κB, and the ratio of p‑JNK/JNK (*p* < 0.001, *p* < 0.05, *p* < 0.05, respectively) further decreased in the group pretreated with siWnt5a and alprostadil compared with the LPS+siWnt5a group.

Based on these results, it was considered that alprostadil alone had no significant effect on the Wnt5a/JNK/NF-κB signaling pathway. The Wnt5a/JNK/NF-κB pathway was activated by LPS and inhibited by pretreatment with alprostadil in a similar way to siWnt5a. Besides, alprostadil combined with siWnt5a exerted a synergistic inhibitory effect on the Wnt5a/JNK/NF-κB signaling pathway activated by LPS. SP60025 was used to further investigate the underlying mechanisms of alprostadil inhibition of Wnt5a/JNK/NF-κB signaling in LPS-induced cardiomyocytes. We found that the expression of Wnt5a, NF-κB, and the ratio of P‑JNK/JNK were significantly decreased in the LPS+SP60025 group (*p* < 0.01, *p* < 0.05, *p* < 0.01, respectively) compared with the LPS group, and their expression was further decreased in the group pretreated with SP600125 and alprostadil (*p* < 0.01, *p* < 0.01, *p* < 0.01, respectively) compared with the LPS+SP600125 group. Interestingly, it was found that the NF-κB level was higher in the LPS+SP60025 group than that in the LPS+siWnt5a group and the LPS+siWnt5a+alprostadil group (*p* < 0.05), indicating that SP600125 inhibited the Wnt5a/JNK/NF-κB signaling pathway and showed a synergistic effect with alprostadil. Phosphorylated JNK significantly promoted NF-κB nuclear entry, which might have a crosstalk effect with other signaling pathways.

## Discussion

Sepsis is a systemic inflammatory response syndrome (SIRS) usually caused by endotoxin [30]. Myocardial injury, known as septic cardiomyopathy (SC), is one of the most serious complications of sepsis. The exact mechanism of SC remains unclear; it has been found that endotoxin and inflammatory molecules may be responsible for myocardial injury, derangement in cardiomyocyte physiology at the microcirculatory level, mitochondrial dysfunction, disruption of normal calcium handling, and autophagy deficiency [4, 5, 31]. The conventional therapy for septic cardiomyopathy includes fluid resuscitation and administration of vasopressors. Cardiac inotropes such as levosimendan, a calcium sensitizer, may improve myocardial contractibility in the absence of increased oxygen consumption and may also be useful for septic cardiomyopathy [32], but this view remains controversial [33]. Other studies have shown that veno-arterial extracorporeal membrane oxygenation (ECMO) or intra-aortic balloon pumping (IABP) was beneficial for patients with severe septic cardiomyopathy [34, 35]. Some studies reported that the cardiac function of patients suffering from sepsis could recover fully to the premorbid state [36, 37] and the reversibility in SC may be similar to myocardial ischemia preconditioning [38, 39]. Honda et al. found that remote ischemic conditioning (RIC), a highly cardioprotective phenomenon induced by repeated transient ischemia of a remote organ or tissue, reduced circulating inflammatory mediators associated with septic cardiomyopathy, suppressed inflammatory signaling pathways in heart tissue, reduced cardiac damage, and consequently preserved ventricular function in LPS-induced septic cardiomyopathy [40]. In this study, LPS was used as a stimulus to induce inflammatory damage in H9c2 cells and to establish the SC cell model as described previously [41]. It was observed that the cell viability gradually decreased after treatment with LPS in a dose-dependent and time-dependent manner, in which high concentrations of LPS (100 μg/l) significantly increased myocardial cell injury markers such as LDH and troponin as well as inflammatory factors such as IL-1, IL-6, IL-17, and TNF-α in myocardial cells. These results indicate that LPS led directly to cell injury in a dose-dependent manner.

Wnt5a, a secretory glycoprotein that is mainly involved in nonclassic pathways, is an inflammatory response molecule derived from macrophages. It can stimulate the release of other inflammatory response factors through autocrine or paracrine signaling [42]. Moreover, Wnt5a activates the downstream JNK/NF-κB signaling pathway, is involved in crosstalk with other signaling pathways, and plays a role in cellular inflammatory responses. Wnt5a can be induced by LPS/IFN-γ in human macrophages [11–13]. In our study we also found that LPS induced H9c2 cells to secrete Wnt5a, elevated the JNK phosphorylation level, and increased the amount of NF-κB in the nuclei, which was reversed by the application of siWnt5a. These results suggest that LPS could activate the Wnt5a/JNK/NF-κB pathway in H9c2 cells. Pretreatment with SP60025 significantly reduced the amount of NF-κB in the nucleus, which, however, was still higher than in the siWnt5a group. These results suggest that NF-κB may also be regulated by other signaling pathways besides JNK.

Alprostadil can protect the microcirculation and inhibit inflammatory responses, and, as is known, have anti-inflammatory and anti-apoptotic effects on the myocardium [24–27]. We found that alprostadil reversed the decline of cell activity, reduced LDH and troponin levels, and down-regulated the expression of inflammatory factors in H9c2 cells treated with LPS, which suggests that alprostadil had a protective effect on myocardial cells. Following this, we explored its mechanisms. The expression levels of Wnt5a, NF-κB, and p‑JNK/JNK did not change significantly when treated with alprostadil alone, indicating that alprostadil did not have significant influence on the Wnt5a/JNK/NF-κB signaling pathway in cardiac cells without LPS stimulation. However, it was observed that alprostadil decreased the mRNA levels of Wnt5a, JNK, and NF-κB in H9c2 cells treated with LPS, as well as the protein levels of Wnt5a, p‑JNK/JNK, and NF-κB in the nucleus, respectively, indicating that alprostadil had an inhibitory effect on the Wnt5a/JNK/NF-κB pathway activated by LPS. Next, H9c2 cells was pretreated with siWnt5a, and it was found that siWnt5a had a similar inhibitory effect on the Wnt5a/JNK/NF-κB signaling pathway to alprostadil. These results indicate that alprostadil might play a role by inhibiting Wnt5a in the Wnt5a/JNK/NF-κB pathway. Moreover, it was found that alprostadil combined with siWnt5a or SP60025 could further inhibit the Wnt5a/JNK/NF-κB pathway activated by LPS, thereby playing a cellular protective role. Collectively, it was demonstrated that alprostadil exerted its potential protection against LPS-induced injury of H9c2 cardiomyocytes via the Wnt5a/JNK/NF-κB pathway.

## Conclusion

The application of alprostadil can reduce the increase in cytokine levels and cell damage caused by lipopolysaccharides in H9c2 cardiomyocytes. The anti-inflammatory effects may be generated by inhibiting the Wnt5a/JNK/NF-κB pathway.
